# Numerical optimization of gene electrotransfer into muscle tissue

**DOI:** 10.1186/1475-925X-9-66

**Published:** 2010-11-04

**Authors:** Anze Zupanic, Selma Corovic, Damijan Miklavcic, Mojca Pavlin

**Affiliations:** 1 University of Ljubljana, Faculty of Electrical Engineering, Trzaska cesta 25, SI-1000 Ljubljana, Slovenia

## Abstract

**Background:**

Electroporation-based gene therapy and DNA vaccination are promising medical applications that depend on transfer of pDNA into target tissues with use of electric pulses. Gene electrotransfer efficiency depends on electrode configuration and electric pulse parameters, which determine the electric field distribution. Numerical modeling represents a fast and convenient method for optimization of gene electrotransfer parameters. We used numerical modeling, parameterization and numerical optimization to determine the optimum parameters for gene electrotransfer in muscle tissue.

**Methods:**

We built a 3D geometry of muscle tissue with two or six needle electrodes (two rows of three needle electrodes) inserted. We performed a parametric study and optimization based on a genetic algorithm to analyze the effects of distances between the electrodes, depth of insertion, orientation of electrodes with respect to muscle fibers and applied voltage on the electric field distribution. The quality of solutions were evaluated in terms of volumes of reversibly (desired) and irreversibly (undesired) electroporated muscle tissue and total electric current through the tissue.

**Results:**

Large volumes of reversibly electroporated muscle with relatively little damage can be achieved by using large distances between electrodes and large electrode insertion depths. Orienting the electrodes perpendicular to muscle fibers is significantly better than the parallel orientation for six needle electrodes, while for two electrodes the effect of orientation is not so pronounced. For each set of geometrical parameters, the window of optimal voltages is quite narrow, with lower voltages resulting in low volumes of reversibly electroporated tissue and higher voltages in high volumes of irreversibly electroporated tissue. Furthermore, we determined which applied voltages are needed to achieve the optimal field distribution for different distances between electrodes.

**Conclusion:**

The presented numerical study of gene electrotransfer is the first that demonstrates optimization of parameters for gene electrotransfer on tissue level. Our method of modeling and optimization is generic and can be applied to different electrode configurations, pulsing protocols and different tissues. Such numerical models, together with knowledge of tissue properties can provide useful guidelines for researchers and physicians in selecting optimal parameters for *in vivo *gene electrotransfer, thus reducing the number of animals used in studies of gene therapy and DNA vaccination.

## Background

In the last decades advances in genetic research offered a set of new therapies for various diseases based on *in vivo *genetic manipulations. The most developed of them are gene therapy and DNA vaccination, which have already been tested in several clinical trials [1-4]. While gene therapy works by delivering therapeutic genes into target cells to express themselves and produce proteins acting directly against a given disease, in genetic vaccination the produced proteins act as antigens that illicit an immune response [5,6]. In the future, genetic therapies could represent an effective treatment for degenerative diseases, cancer, infections and cardiovascular diseases, for which currently no adequate treatments are available [3,7-10].

The first step for gene therapy and DNA vaccination is efficient transfer of DNA molecules into target cells. *In vitro*, chemical, physical and biological methods have been successfully used for gene transfer [7,11,12]. However, there have been difficulties of translating these methods into *in vivo *settings. Currently, viral vectors boast the highest transfection efficiency, but this efficiency comes with an increased risk of viral infection [13,14]. Therefore, alternative methods are being developed. One of the most promising physical methods for gene transfer *in vivo *is gene electrotransfer which, in comparison to viral vectors, is not hampered in terms of immunogenicity or pathogenicity. Gene electrotransfer combines the use of pDNA and local application of electric pulses, which increase the permeability of target cells (electroporation) for different molecules, including pDNA, and thus enable transfer of DNA into the cell. Compared to other physical and chemical methods, it was shown that gene electrotransfer is the most versatile and also the most efficient method *in vivo *compared to other methods. For example, the gene gun method is limited to exposed tissues while complexes of DNA and cationic lipids or polymers can be unstable, inflammatory and toxic [7]. Gene electrotransfer has therefore great potential to be used in clinics for treatment of cancer and various chronic diseases [15-22], and also for DNA vaccination for prevention of various infectious diseases and HIV [6]. Moreover, recently it was demonstrated that gene electrotransfer can be successfully applied as a method for DNA vaccination for cancer treatment [6,23,24], where DNA for a certain tumor antigen is transferred during remission and can thus prepare the immune system for a better response against the tumor cells during relapse of the disease.

Gene electrotransfer was demonstrated almost 30 years ago [25] when it was first shown that exposing cells to high-voltage electric pulses results in transfer of DNA molecules and expression of the delivered genes. Up to now, several steps that are involved in gene electrotransfer have been identified: electropermeabilization of the cell membrane, contact of pDNA with the cell membrane (formation of a DNA-membrane complex), translocation of pDNA across the membrane, transfer of pDNA to and into the nucleus and gene expression [26-29]. The effectiveness of electroporation and consequently of gene electrotransfer depends on pulse parameters, such as amplitude, duration, number, pulse repetition frequency and geometric properties of electrode and tissue/sample configuration [30-33]. These parameters define the duration of exposure to external electric field and the electric field strength, which have been shown to be the most important parameters in cell electroporation. Namely, molecular transport into and out of cells is observed only above a threshold value for reversible electroporation *E > E_rev_*. When electric field is further increased above the irreversible electroporation threshold *E *>*E_irr _*(or when longer and/or several pulses are used) the changes in the cell membrane become irreversible and cells die. Therefore for efficient gene electrotransfer it is crucial to choose an appropriate applied voltage/electrode configuration, such that the local electric field in the target tissue is between the reversible and irreversible electroporation threshold *E_rev _> E > E_irr_*, which enables gene transfer and preserves cell viability, thus enabling successful gene expression.

Gene electrotransfer was first demonstrated *in vivo *in 1998 by several independent studies [34-37]. It is currently being extensively studied on animal models *in vivo *for gene therapy [7,16,19,30,38,39] as well as DNA vaccination [6,23,40]. In the last years the first human clinical trials have also started and also show encouraging results [18,19,41-43]. One of the major obstacles towards translating gene electrotransfer into clinical applications is its relatively low efficiency. Even though it was demonstrated that in skin [39,44-50] and muscle tissue [34,37,51-56] prolonged expression of transfected genes can be achieved, the relative transfection rates have remained relatively low. In order to improve transfection efficiency the parameters of electric pulses have to be optimized depending on the type of electrodes used and specifically adjusted for each target tissue (e.g. muscle, skin, tumor tissue).

Several researchers have demonstrated that numerical modeling can be used to predict the extent of electroporation in biological tissues [57-64]. Also, numerical modeling and optimization have already been used for optimization of electric pulse parameters for electrochemotherapy of subcutaneous tumors, for tumor ablation with irreversible electroporation [63,65-67] and recently, the first deep-seated tumor was treated with electrochemotherapy based on a numerical treatment plan [68]. However, up to now there exists no such study which would use numerical modeling for optimization of gene electrotransfer.

In our present study we used 3D numerical modeling and numerical optimization to determine the best electrical parameters for efficient gene electrotransfer into muscle tissue, which is regarded by many as the "tissue of choice" for gene electrotransfer-based gene therapy and DNA vaccination [19,30,41,69,70]. We performed a parametric study to better understand how various electroporation parameters (applied voltage, number of electrodes used, electrode positions, insertion depth) affect the electric field distribution in muscle tissue, and numerical optimization of the parameters to demonstrate that such numerical "treatment planning" could be used by researchers and clinicians to better control the extent of electroporation in the target tissues. We compared different needle electrode configurations recommended in the literature for gene electrotransfer in large animals and humans and analyzed the effect of orientation of the electrodes (and thus the electric field) with respect to the orientation of muscle fibers. The methods used in this study and the obtained results can be used as guidelines for future numerical studies and planning of gene electrotransfer *in vivo*.

## Methods

### Model geometry and tissue properties

**Figure 1:**
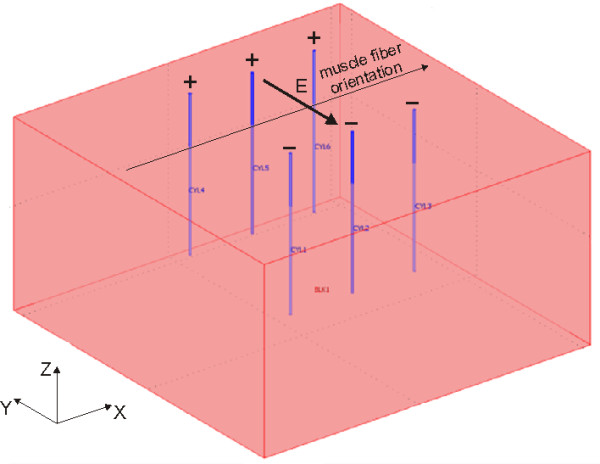
**Model geometry with muscle tissue (pink) and needle electrodes (blue)**.

The numerical model of electroporation used in our study was similar to the one used by Corovic *et al *for modeling electroporation of muscle tissue in small animals [71]. In short, muscle tissue geometry was modeled as a block of size 10 × 10 × 6 cm in the direction of the X, Y and Z axis, respectively (Figure 1), with the long axis of the muscle fibers aligned with the × axis. The size chosen is similar to the size of a larger human muscle and at the same time represents a sufficiently large computational domain to avoid any significant numerical errors due to boundary conditions. Two different needle electrode configurations were used in our model: two needle electrodes and six needle electrodes arranged into two rows of three electrodes (Figure 1). The needle electrodes were modeled as 5 cm long stainless steel cylinders with diameters of 0.7 mm.

**Figure 2:**
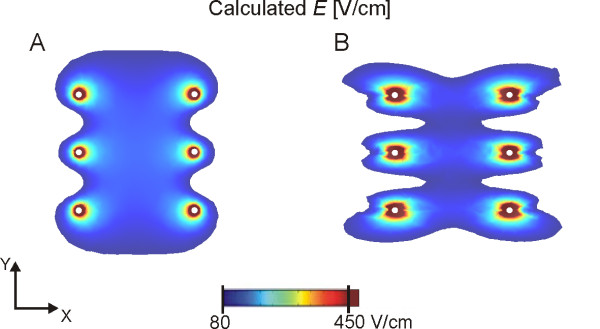
**Electric field distribution for isotropic and anisotropic muscle conductivity**. Electric field distribution for six needle electrodes (white circles) with a) isotropic tissue and b) anisotropic tissue properties is shown. Electric field between 80 V/cm and 450 V/cm are presented in colors from blue to red, while fields over 450 V/cm are presented in dark brown (around the electrodes).

The muscle tissue was considered anisotropic, with higher conductivity in the direction parallel to muscle fibers (*σ^1^_xx _*= 0.75 S/m; *σ^2^_xx _*= 2.0 S/m) than in the perpendicular direction (*σ^1^_yy _*= *σ^1^_zz _*= 0.135 S/m; *σ^2^_yy _*= *σ^2^_zz _*= 0.54 S/m). Index ^1 ^denotes initial values prior to electroporation, while index ^2 ^denotes the maximum achieved conductivities in the model (after irreversible electroporation is achieved in the tissue) [72]. The reversible electroporation threshold values for muscle tissue were taken to be 80 V/cm and 200 V/cm for electric field parallel and perpendicular to muscle fiber orientation, respectively, while the irreversible threshold was taken to be 450 V/cm irrespective of electric field direction. These values were selected considering both our previous studies of *in vivo *electroporation studies [71,73], where the thresholds were measured for 8 × 100 μs electric pulses, and the measurements of muscle tissue conductivity found in the available literature [74]. The importance of using anisotropic tissue properties instead of isotropic properties is illustrated in Figure 2, where the difference in electric field distribution between both cases is clearly seen.

### Numerical modeling

The numerical models were designed in Comsol Multiphysics 3.5a (Comsol AB, Sweden) and solved with the finite element method on a desktop PC (Windows 7 64-bit, Intel Core 2 Duo 2.66 GHz, 4 GB RAM). The electric field distribution was determined by solving the Laplace equation for static electric currents:

(1) ∇(σ(−∇u))=0,

where *σ *is the tensor of electrical conductivity and *u *the electric potential. The boundary conditions used in our calculations were: 1) constant potential on the surface of the active parts of the electrodes and 2) insulation (***n·J ***= 0) on the outer boundaries of the model.

As the parametric study and optimization described in the next section involved moving and rotating the electrodes with respect to the muscle tissue and thus repeated meshing of the model geometry, some of the meshing could be avoided by rotating the tissue properties (thus virtually rotating the muscle tissue) instead of the electrodes. The final form of the conductivity used in the models is therefore given by a tensor:

(2) $$\sigma =\left(\begin{array}{ccc} \sigma_{xx}\sin^{2}\phi +\sigma_{yy}\cos^{2}\phi & \left|\sigma_{xx}-\sigma_{yy}\right|\sin\phi \cos\phi & 0\\ \left|\sigma_{xx}-\sigma_{yy}\right|\sin\phi \cos\phi & \sigma_{yy}\sin^{2}\phi +\sigma_{xx}\cos^{2} & 0\\ 0 & 0 & \sigma_{zz} \end{array}\right)$$

Since tissue properties are known to change during electroporation [72,75,76], each component (*σ_xx _*and *σ_yy_= σ_zz_*) of electrical conductivity was modeled as an electric field-dependent function *σ(E)*:

(3) $$\sigma \left(E\right)=\frac{\sigma_{2}-\sigma_{1}}{E_{irr}-E_{rev}}\cdot E+\sigma_{1},$$

where *σ_1 _*and *σ_2 _*are tensors of electrical conductivities of non-electroporated and electroporated tissues, respectively (see previous section), and *E_irr _*and *E_rev _*are the thresholds of irreversible and reversible electroporation, respectively. We approximated the dynamics of the conductivity changes during electroporation by performing several sequential calculation of the electric field distribution, while changing the conductivities according to Eq. 3. The details of the sequential analysis can be found in our previous work [57,73].

The results of the numerical modeling were analyzed by calculating the volumes for reversibly and irreversibly electroporated muscle tissue, *V_rev _*and *V_irr_*, respectively, and total current through the tissue *I*. *V_rev _*was calculated by integrating the volume of muscle tissue, where conductivity has changed (*σ_xx_*, *σ_yy _*or *σ_zz_*), while *V_irr _*by integrating the volume, where the electric field was over the irreversible electroporation threshold *E > E_irr_*.

### Parametric study

**Figure 3:**
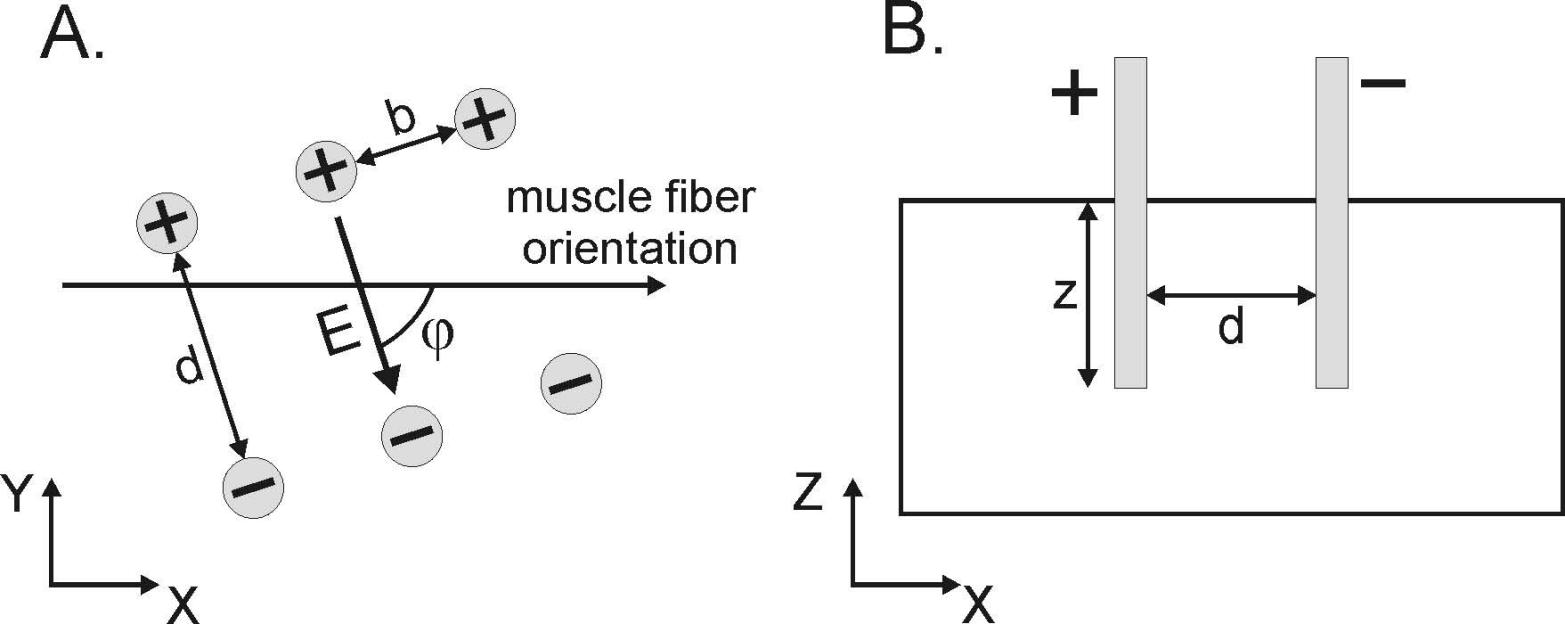
**Geometrical and electrical parameters analyzed in the parametric and optimization study**. Two electrodes and six electrodes in muscle tissue in A) the XY plane and B) the XZ plane are shown. The following parameters were optimized: *d *(4 mm and 8-56 mm, 8 mm step); *b *(4-28 mm, step of 4 mm); *z *(10-40 mm; 10 mm step); *ϕ *(0-90 °; 22.5° steps) and *U *(400-2400 V, 200 V step for six electrodes; 600-3000 V for two electrodes). Note that *ϕ *= 0° is referred to in the text as the parallel orientation, while *ϕ *= 90° is referred to as the perpendicular orientation.

To determine the best electrode positions and voltages between electrodes for gene electrotransfer into muscle tissue several geometrical and electrical parameters were analyzed in a parametric study. Bound constraints for each parameter and discretization steps were chosen as following: distance between electrodes of different polarity - *d *(4 mm and 8-56 mm, 8 mm step); distance between electrodes of the same polarity - *b *(4-28 mm, step of 4 mm); depth of electrode insertion - *z *(10-40 mm; 10 mm step); angle between the electric field and muscle fiber orientation - *ϕ *(0-90°; 22.5° steps) and voltage between the electrodes - *U *(400-2400 V, 200 V step for six electrodes; 600-3000 V for two electrodes) (Figure 3). Altogether 12,320 calculations were performed for six electrodes and 2,080 for two electrodes. The ranges of geometric parameters were selected to scale from typical dimensions used for gene electrotransfer in small animals to dimension applicable to large animals and humans. Results were controlled for numerical errors by increasing the size of our model domain and increasing the mesh density, until error due to domain size and due to meshing irregularities were insignificant--a further increase in domain size or mesh density only increased the computation time; however, the results (*V_rev_*, *V_irr_*) changed less than 2%. The quality of solutions for given sets of parameters was evaluated by calculating the volume of muscle tissue that was reversibly but not irreversibly electroporated, and the electric currents flowing through the model, as presented in the objective function:

(4) $$F=\left\{\begin{array}{cc} 0, & \text{if}\left(V_{irr}>1\;{\text{cm}}^{3}|I>30\;\text{A}\right)\\ V_{rev}-V_{irr,} & \text{otherwise} \end{array}\right\}.$$

The limit value for *V_irr _*(1 cm^3^) was based on our estimate of tissue damage produced by electrode insertion and the fact that some tissue damage is always present around needle electrodes during electroporation. The limit value for I (30 A) was based on the limitation of electroporation devices available on the market at the time of the study. By taking into account constraints for *V_irr _*and for *I*, extensive damage to muscle tissue is avoided and compliance with electric pulse generator limitations guaranteed.

### Optimization

The same parameters (distances between electrodes, depth of insertion, voltage between electrodes) and the same objective function used in the parametric study were also used in the optimization, only the steps were smaller: *d *- 2 mm, *b *- 2 mm, *z *- 5 mm, *ϕ *- 10° and *U *- 100 V. For the optimization we used a genetic algorithm that has been described in detail in our previous work [66]. In short, the genetic algorithm was written in MATLAB 2007a (Mathworks, USA) and run together with the numerical calculation using the link between MATLAB and COMSOL. The initial population of chromosomes (vectors of real numbers - one for each optimized parameter) was generated randomly, taking into account the bound constraints. In each iteration, the chromosomes were selected for reproduction with a probability proportional to the values of their objective function. The selected chromosomes reproduced by mathematical operations of crossover (5) or mutation (6):

(5) $$\begin{array}{cc} z_{i}=a_{i}\cdot x_{i}+\left(1-a_{i}\right)y_{i}, & a_{i}\in \left[0,1\right] \end{array},$$

(6) $$\begin{array}{cc} m_{i}=x_{i}+b_{i}\cdot x_{i}, & b_{i}\in \left[-0.3,0.3\right], \end{array}$$

where *z_i _*and *m_i _*are child chromosomes, *x_i _*and *y_i _*are parent chromosomes and *a_i _*and *b_i _*are numbers randomly chosen from the given intervals. The optimization was stopped after the chromosome with the highest objective function value has not improved in 20 iterations for more than 0.1%, which was interpreted as reaching a solution very close to the global optimum.

## Results

### Parametric study

**Figure 4:**
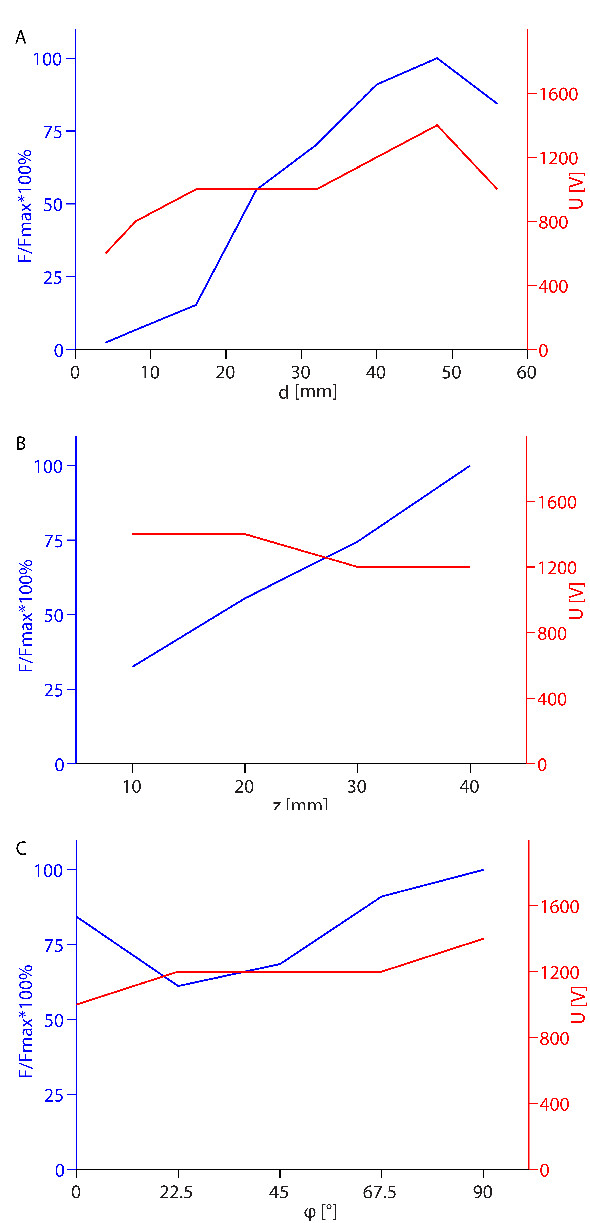
**Optimal values of the objective function depending on geometrical parameters for two electrodes**. Left axis: maximum values of obtained objective functions normalized by the absolute maximum value of objective function obtained in the parametric study (*F/Fmax*); right axis: voltage between electrodes (*U*) that leads to the maximum values. All the figures show optimal solutions for each given value of the parameter shown on the horizontal axis, while the other parameters change in steps as defined in caption of Figure 3 for two electrodes. *F/Fmax *is presented in dependence of: A) the distance between electrodes - *d*, B) depth of electrode insertion - *z *and C) angle of electric field with respect to muscle fiber orientation - *ϕ*.

**Figure 5:**
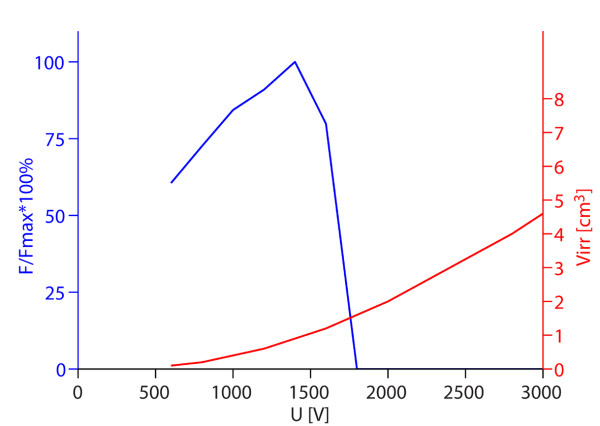
**Optimal values of the objective function depending on voltage between electrodes for two electrodes**. Left axis: maximum values of obtained objective functions normalized by the absolute maximum value of objective function obtained in the parametric study (*F/F_max_*); right axis: average volume of irreversible electroporation obtained by using those voltages (*V_irr_*). The figure shows optimal solutions for given values of *U*, while the other parameters change in steps as defined in caption of Figure 3 for two electrodes.

**Figure 6:**
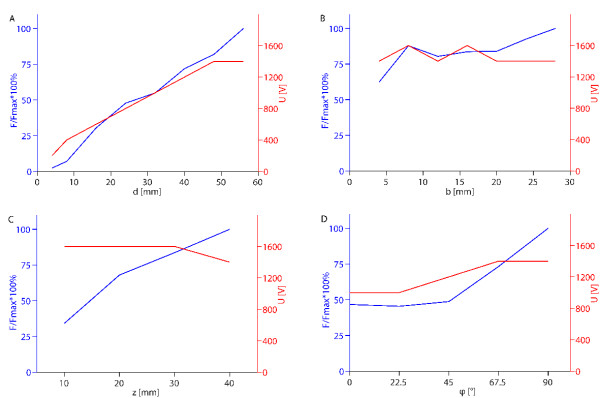
**Optimal values of the objective function depending on geometrical parameters for six electrodes**. Left axis: maximum values of obtained objective functions normalized by the absolute maximum value of objective function obtained in the parametric study (*F/F_max_*); right axis: voltages between rows of electrodes (*U*) that lead to the maximum values. All the figures show optimal solutions for each given value of the parameter shown on the horizontal axis, while the other parameters change in steps as defined in caption of Figure 3 for six electrodes. *F/F_max _*is presented in dependence of: A) the distance between rows of electrodes - *d*, B) distance between electrode in a row - *b*, C) depth of electrode insertion - *z *and D) angle of electric field with respect to muscle fiber orientation - *ϕ*.

**Figure 7:**
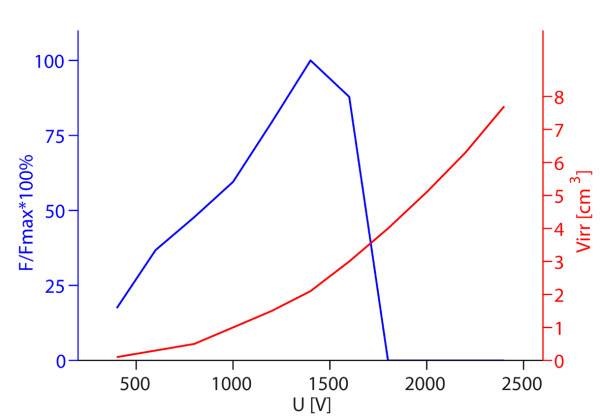
**Optimal values of the objective function depending on voltage between electrodes for six electrodes**. - Left axis: maximum values of obtained objective functions normalized by the absolute maximum value of objective function obtained in the parametric study (*F/F_max_*); right axis: average volume of irreversible electroporation obtained by using those voltages (*V_irr_*). The figure shows optimal solutions for given values of *U*, while the other parameters change in steps as defined in caption of Figure 3 for six electrodes.

The parametric study produced a vast number of different solutions of electric field distribution (2,080 for two needle electrodes and 12,320 for six needle electrodes), which were analyzed by calculating the volumes of reversibly and irreversibly electroporated tissue, and the objective function value (Eq. 4). Figures 4, 5, 6 and 7 show the optimal of these solutions (highest *F*) for each given value of the analyzed parameter, while all the other parameters change according to the selected bounds and steps (Figure 3). E.g., in Figure 4a for each value of distance between electrodes *d*, all the other parameters (*z*, *ϕ*, *U*) are varied and the optimal solution (highest *F*) is presented at given *d*.

#### Two needle electrodes

For two needle electrodes, increasing the distance between the electrodes (*d*) produces higher values of the objective function up to *d *= 48 mm, however to achieve this, higher voltages have to be used (Figure 4a). In fact, an almost 50-fold increase in the value of objective function is achieved by increasing *d *from 4 mm to 48 mm. At *d *= 56 mm a significant drop in *F *was obtained. In Table 1 the optimal parameters for different distances between the electrodes are presented, together with the calculated total current through the model (*I*) and the volumes of reversibly and irreversibly electroporated tissue (*V_rev _*and *V_irr_*).

**Table 1:** Optimum gene electrotransfer parameters (*z, ϕ*, *U*) for two electrodes.

*d *[mm]	*z *[mm]	*φ *[°]	*U *[V]	*I *[A]	*V_rev _*[cm^3^]	*V_irr _*[cm^3^]
4	20	90	600	26.4	1.2	0.2
8	10	90	800	13.0	4.4	1.0
16	30	90	1000	7.5	8.7	0.9
24	40	90	1000	15.0	28.7	1.0
32	40	90	1000	16.6	36.2	0.7
40	40	90	1200	18.4	46.9	0.8
48	40	90	1400	18.4	51.5	0.9
56	40	0	1000	18.9	43.4	0.8

Calculated values of *I, V_rev_, V_irr _>*determined by the parametric study for different distances between electrodes (*d*).

Increasing the depth of insertion (*z*) also produces higher objective function values, with Figure 4b suggesting a linear relationship between the two. Positioning the electrodes so that the electric field is perpendicular to the direction of muscle fibers produces objective functions up to 20% higher than the parallel orientation (Figure 4c), however slightly higher voltages had to be used to achieve this.

Figure 5 shows how increasing the voltage between electrodes affects the objective function. At lower voltages increasing the voltage increases the objective function, however after a peak is reached (1400 V) the objective function values start to sharply decrease, because the damage to tissue exceeds the values (*V_rev _*or *I*) tolerated by the objective function.

#### Six needle electrodes

When we analyzed solutions for six electrodes (Figure 6) similar results were obtained as for two electrodes. Increasing the distances between electrodes and depth of insertion leads to higher values of the objective function and demands higher voltages (Figure 6a, c). Table 2 shows how changing the distance between the electrode rows affects the other parameters needed to achieve the highest values of the objective function and *V_rev_*. Increasing the distance between electrodes in a row (*b*) also produces some effect, but mainly only between positioning the electrodes very close together or very far apart (Figure 6b). Furthermore, we obtained a significant effect of the electric field orientation on the quality of the solution. Positioning the electrode perpendicularly to the direction of muscle fiber (*ϕ = 90°*) produced solution with twice higher values of the objective function (Figure 6d) compared to parallel orientation of the electrodes (*ϕ = 0°*).

**Table 2:** Optimum gene electrotransfer parameters (*b, z, ϕ*, *U*) for six electrodes.

*d *[mm]	*b *[mm]	*z *[mm]	*φ *[°]	*U *[V]	*I *[A]	*V_rev _*[cm^3^]	*V_irr _*[cm^3^]
4	8	30	90	200	23.3	3.7	0.1
8	24	20	90	400	18.1	12.0	0.1
16	24	40	90	600	29.6	49.0	0.8
24	24	40	90	800	29.9	76.5	0.8
32	16	40	90	1000	28.5	87.5	0.7
40	16	40	90	1200	29.8	114.6	0.8
48	24	30	90	1400	27.4	130.6	0.9
56	28	40	90	1400	28.5	158.9	0.6

Calculated values of *I, V_rev_, V_irr _>*determined by the parametric study for different distances between electrodes (*d*).

Figure 7 shows how increasing the voltage between the electrode rows affects the objective function: at lower voltages increasing the voltage increases the objective function, however after the maximum is reached (1400 V) the objective function values sharply decrease.

### Optimization

**Figure 8:**
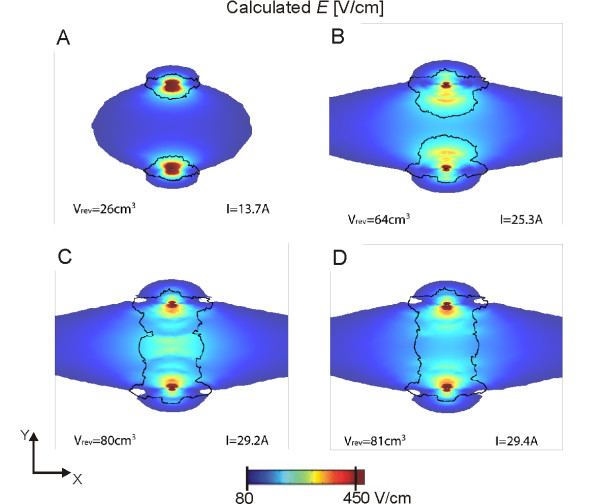
**Four steps of the sequential analysis (A-D) of the electroporation process for two electrodes**. The optimum parameters as determined by the genetic algorithm were: *d = 56 mm*, *z = 40 mm, ϕ = 90°, U = 1550 V*. The electric field distribution is shown for a plane perpendicular to electrode insertion 2 cm deep in muscle tissue. The black contour represents the muscle area that has been reversibly electroporated, i.e. area where the conductivity values have changed according to (3). Due to ratio problems, the contour for irreversibly electroporated muscle tissue is left out of the figure; however it is approximately the same as the dark red coloration surrounding the electrodes in sequence D.

**Figure 9:**
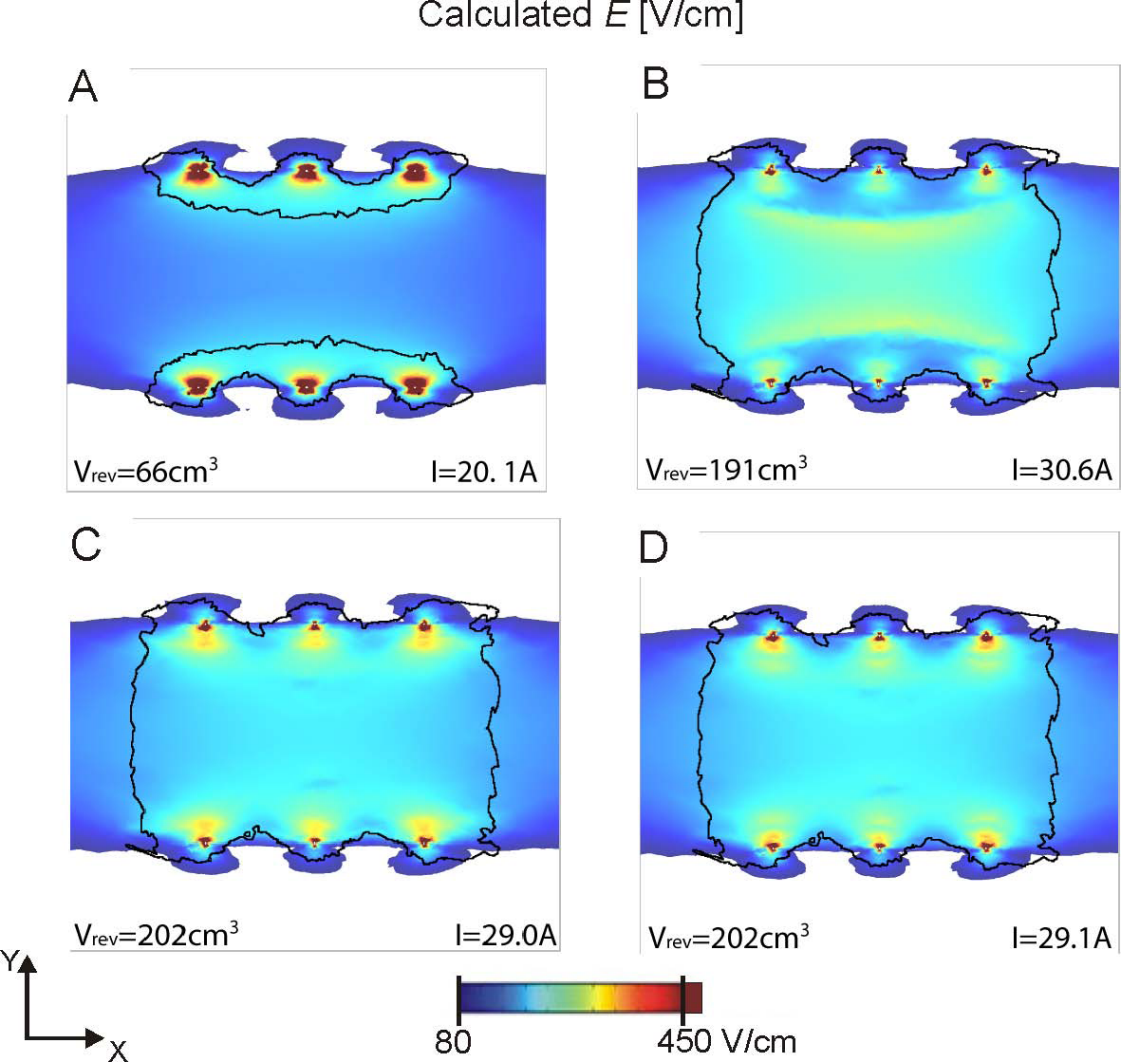
**Four steps of the sequential analysis (A-D) of the electroporation process for six electrodes**. The optimum parameters as determined by the genetic algorithm were: *d = 56 mm*, *b = 28 mm, z = 40 mm, ϕ = 90°, U = 1350 V*. The electric field distribution is shown for a plane perpendicular to electrode insertion 2 cm deep in muscle tissue. The black contour represents the muscle area that has been reversibly electroporated, i.e. area where the conductivity values have changed according to (3). Due to ratio problem, the contour for irreversibly electroporated muscle tissue is left out of the figure, however it is approximately the same as the dark red coloration surrounding the electrodes in sequence D.

**Figure 10:**
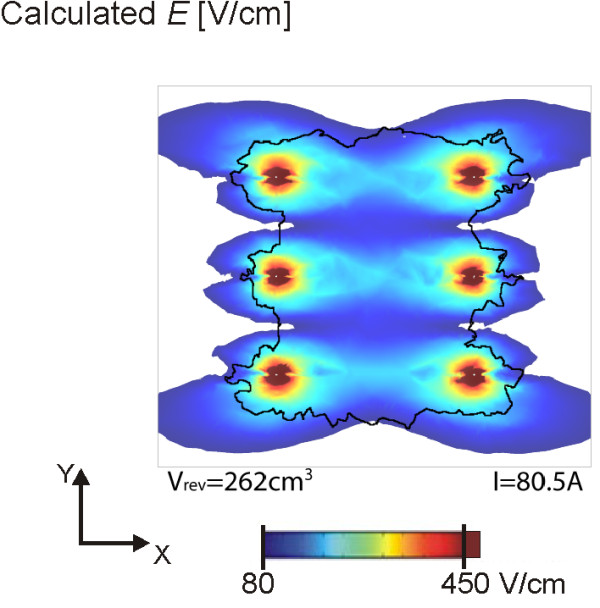
**The last step of the sequential analysis of the electroporation process for six electrodes**. The same parameters as in Figure 9 were used, except for *ϕ = 0°*. The electric field distribution is shown for a plane perpendicular to electrode insertion 2 cm deep in muscle tissue. The black contour represents the muscle area that has been reversibly electroporated, i.e. area where the conductivity values have changed according to (3). Due to ratio problem, the contour for irreversibly electroporated muscle tissue is left out of the figure; however it is approximately the same as the dark red coloration surrounding the electrodes.

The optimization results for two electrodes are presented in Figure 8 for 4 sequences of the calculation, taking into account the changes in conductivity during electroporation. The first sequence (Figure 8A), which matches the calculation performed with a static model of electroporation (no changes in conductivity), shows that at the beginning of the electric pulse the volume of reversibly electroporation (black contour in Figures 8, 9 and 10) is mostly located around the electrodes. In the next sequences one can see that the increase of conductivity due to electroporation extends the higher electric fields towards the area in the center between the electrodes, resulting in much larger volumes of reversibly electroporated muscle tissue (26 cm^3 ^vs. 81 cm^3^). This results in a very large volume of reversible electroporation and a much lower volume of irreversible electroporation (0.9 cm^3^). It is somewhat surprising that the perpendicular direction of the electric field produced better results, since the threshold for electroporation is higher in this direction and therefore lower volumes would be expected. This can be explained by a much higher current that is generated by the field parallel to muscle fiber direction (conductivity is higher in that direction), thus the limitation for electric current (30 A) set in the objective function are exceeded earlier for the parallel direction.

The optimization results for six electrodes are presented in Figure 9. In this case, the perpendicular orientation produced almost two times better results than the parallel orientation. Similarly as for two electrodes, the sequence of images in Figure 9 shows, how taking into account the changes in conductivity during electroporation increases the calculated volumes of reversible electroporation. When analyzing how *V_rev _*increases with consecutive steps we obtained that it is a highly non-linear function where in the first step the biggest increase occurs; for two electrodes the increase is 2.5 times after the first step and 3.1 times at the final sequence; for six electrodes the increase in *V_rev _*after the first step is 2.9 times and 3.1 at the end of the sequence. The changes in *V_irr _*are negligible due to limitation of *V_irr _*< 1 cm^3^.

The difference between the perpendicular and parallel orientation can be illustrated by using the same parameters as determined for optimal solution shown in Figure 9, but by changing the direction of the generated electric field so that it is aligned with muscle fibers (*ϕ = 0°*). In this case, even more muscle volume becomes reversibly electroporated, however at the same time, the current flowing through the tissue (80.5 A) is much higher than the allowed current (30A) and also much higher compared to the current for the perpendicular orientation (29.1 A). Also approximately three times more tissue is irreversibly electroporated for parallel compared to perpendicular orientation (Figure 10).

## Discussion

Gene electrotransfer is already successfully being used for pDNA delivery in clinical applications, such as gene therapy and DNA vaccination. However, achieving an adequate extent of electroporation in target tissues can be difficult and requires extensive experimentation. Numerical modeling of electroporation can be used to complement in vivo experimentation as it allows planning of the electric field distribution beforehand. In this study we used 3D numerical modeling of muscle tissue, parameterization and numerical optimization to determine the affects of different geometrical and electrical parameters on the distribution of electric field in muscle tissue and the optimum parameters for gene electrotransfer in muscle tissue.

In the first part of this work we performed a parametric study to determine how various parameters affect the electric field distribution and volumes of tissue exposed to only reversible electric fields in muscle tissue, which is a prerequisite for effective gene electrotransfer. We determined that for large distances between the electrodes (*d*) and for placing the electrodes deeper into the muscle (*z*), the volumes of reversibly electroporated tissue (*V_rev_*) increases. However, concurrently the volume of irreversibly electroporated tissue (*V_irr_*) and total current through the tissue (*I*) also increase (see Tables 1 and 2, and Figures 4 and 6). Increases in *V_irr _*and *I *can be explained with higher voltage needed to reach the optimal electric field distribution at given *d *(Figures 4a and 6a), while for deeper insertion (larger *z*) *V_irr _*and *I *increase due to a larger surface area of the electrodes in the tissue. For two electrodes the limiting factor is *V_irr_*, which increases over the limit of 1 cm^3 ^if voltage over 1600 V is used. For six electrodes *I *increases over 30 A before *V_irr _*gets over 1 cm^3 ^and *I *can therefore be considered the limiting factor. Obviously, by choosing a different objective functions with different limit for *V_irr _*and *I *it is possible to control their importance and thereby adjust the results accordingly to the application and equipment used. The distance between the electrodes in a row (*b*) for six electrodes does not have much effect on *V_rev _*or *V_irr_*, except at very small distances *b*, which, as expected is not optimal since three electrodes with the same electric potential positioned very closely produce an electric field very similar to the one of only one electrode. The advantages of having several electrodes are therefore lost at small distances *b*.

When analyzing the effect of electrode orientation with respect to muscle fibers, we obtained that the perpendicular orientation of the electrodes (*ϕ *= 90°) is better than for the parallel orientation (*ϕ *= 0°) (Tables 1 and 2), since coverage of larger volumes of tissue with electric field above *E_rev _*and below *E_irr _*can be obtained. This can be mostly explained by the smaller volumes of irreversible electroporation achieved in the perpendicular orientation, while at the same time the current (*I*) flowing along the muscle fibers in the parallel orientations is much higher, therefore the current limitation of the objective function is achieved for lower voltages in the parallel orientation. The perpendicular orientation is significantly better for six needle electrodes (*F*_⊥ _≈ 2 × *F*_||_) while for two needle electrodes the difference is not that large (20%). This is in agreement with the in vivo study in mice, where no statistical difference in gene expression was obtained between the perpendicular and parallel orientation for two needle electrodes [34]. If possible, however, it is best to switch orientation of the electric field during the application of electric pulses for more effective gene electrotransfer, as already shown in several studies [46,77,78].

Furthermore, we analyzed the voltage dependence of the optimal solutions *F*(*U*) in parallel with the volume of irreversibly electroporated tissue *V_irr_*(*U*). We obtained that by increasing the applied voltage the volume of only reversibly electroporated tissue increased until an optimal voltage for a given electrode configuration was achieved (Figures 5 and 7). A further increase in the applied voltage lead to excessive tissue damage (large *V_irr_*) and sharp decrease in the objective function value *F*. This strong dependency on pulse amplitude and relatively narrow window of pulse amplitudes for optimal treatment is in accordance with experimental observations [36,58,71,79], where similarly transfection efficiency gradually increased above a threshold voltage, but further increase in voltage lead to a decrease in transfection.

When the results of the parametric study are examined in details, some surprising results can be seen. The relationship between the maximum objective function value and different parameters is not always a smooth curve (e.g. Figure 6b). This is due to an error introduced by relatively large discretization steps: as the objective functions were only evaluated for discrete values of the analyzed parameters some good solutions were missed. The discretization error is also responsible for a "strange" result for d = 56 mm, where in contrast to all other best solutions the parallel orientation produced better results than the perpendicular orientation. Also, for small distances *d *between the electrodes, *I *becomes the major limiting factor and therefore *V_irr _*does not come even close to the limiting value of 1 cm^3 ^set in the objective function. The reason for this seems to be in the extreme non-linearity of the used model. Namely, for smaller electrode distances the electric currents get very high due to small resistance between the electrodes.

The parametric analysis enabled us to understand the relationship between different parameters that can affect the electric field distribution and thus gene electrotransfer. However, in order to optimize these parameters for a given clinical application (treatment planning) such an exhaustive search of the parameter space would take too long and demands very large computer resources. A better approach is, as already demonstrated for electrochemotherapy [67,68] to directly apply an optimization algorithm to determine the optimal parameters. In our study, optimization took only 1.2 hours compared to 23 hours for the parametric study (for two electrodes) and 1.4 hours compared to 4 days for six electrodes. In our optimization study we used the same objective function as in the parametric study, only the parameter stepping was more accurate (see Methods). Therefore, as expected the obtained optimal solutions differ only slightly from the ones obtained in the parametric study (compare Figures 8 and 9 with Tables 1 and 2). This difference can be attributed to a smaller discretization step used in the optimization.

The electric field distributions of the optimal solutions are presented in Figure 8 (two electrodes) and Figure 9 (six electrodes). It can be seen that taking into account dynamic changes of conductivity during electroporation (sequential analysis) has a substantial effect on the final electric field distribution and *V_rev_*. Namely, in the first sequence only a small volume of muscle tissue is reversibly electroporated; while in the final sequence the whole volume between the electrodes reaches *E >E_rev _*(compare Figures 8a, b, c and 8d). This effect is more pronounced for six electrodes (Figures 9a, b, c and 9d). An array of six electrodes also provides a better electric field distribution overall, with higher objective function values (*F_6 _≈ 3 × F_2_*). These results agree with our previous studies of optimization for electrochemotherapy [59,66] that more electrodes enable coverage of larger volumes of tissue with a more homogeneous field. Six electrodes probably represent a good compromise between optimal electric field distributions and still keeping the invasiveness of the procedure relatively low. Nevertheless, using two electrodes can also produce good results, if parameters are select appropriately as already demonstrated [34,80,81].

The objective function used in our study was chosen according to the prerequisite for efficient gene electrotransfer: the cells should only be electroporated reversibly and not irreversibly. Our choice of objective function is also based on the experimental observation that the volume of electrotransfected muscle tissue correlates with the amount of expressed protein [45]. In several studies of gene electrotransfer it was shown that larger transfected volumes are beneficial while for DNA vaccination the transfected volume is probably not such a critical parameter [19]. The optimization and proper selection of objective function has to be done for a specific application together with researchers and physicians in clinics, based on complementary analysis and evaluation of pain and other undesired effects, such as tissue inflammation or thermal damage.

The presented analysis is partially similar to previous studies of optimization of parameters for electrochemotherapy [66-68] or irreversible ablation of tumors (IRE) [61,63] where parameterization and/or optimization was also used to determine optimal electroporation parameters. However, there are two very important differences between our and the previous studies of ECT and IRE. Firstly, we included muscle anisotropy and sequential analysis in our models which was never done before and has also important effect on optimal solutions. Secondly, for ECT the objective function is set so that the most important parameter (weight) is the coverage of the whole tumor with *E *>*E_rev_*, while irreversible electroporation of the tumor is not severely penalized, since the goal of the therapy is tumor death; in EGT, however, it is enough that target tissue is just above the reversible electroporation threshold, while irreversible electroporation is not acceptable since it does not lead to expression of transfected genes. For this reason the additional constrain of *V_irr _*was put into the objective function, which penalizes solutions that would damage the tissue. For IRE a similarly reasoning would lead to another choice of objective function. Another important difference between the three applications is also what we want to treat: in ECT and IRE we want to treat a well defined target tissue and avoidance of electroporating vital organs is crucial, while for EGT of muscle tissue we want to reversibly electroporate large volumes.

In our numerical models joule heating was not analyzed as several studies have already shown that heating due to short 100 μs is negligible [82,83]. Nevertheless, a conservative estimation of the temperature after the electric pulses (see Figure 9 for parameters used), where cooling of tissue due to heat conduction was not taken into account, was below 46 °C in the vicinity of the electrodes and below 38°C in the center between the electrodes, confirming that the proposed pulses would not cause extensive thermal damage. Furthermore, in our case the 30 A limit for total current prevented unwanted thermal damage. However, if longer trains of pulses were applied, e.g. 8 × 10 ms, thermal damage could become a the limiting factor that would have to be taken into account in the objective function.

We analyzed two and six needle electrode configurations, while plate electrodes were not analyzed since they cannot be applied for gene electrotransfer into muscle tissue in large animals and humans [84,85]. Two needle electrodes were analyzed since they are widely used in in vivo gene electrotransfer, while six electrodes were chosen as they enable a more homogeneous electric field inside treated tissue and altogether more optimal solutions. The relatively large electrode distances used in the study were used to extent applicability of our results from small animals to large animals and humans.

One of the limitations of our numerical model is that it performs calculations on a limited domain of muscle tissue and that no other tissues (e.g. skin) are included. This is justified since we checked how the size of our model and boundaries affect the final results. No significant variation of the results (less than 2%) was obtained if the size of the model was increased for a factor of two. Also we have previously shown that if an additional layer with much lower conductivity is added on the top surface (resembling skin tissue) no significant effect is observed on the electric field distribution [66].

We further have to stress that the presented model and optimization was performed for relatively short electric pulses (8 × 100 μs) since the thresholds *E_rev _*and *E_irr _*are relatively well defined [72]. In a variety of studies of in vivo gene electrotransfer it was shown that different electric pulse parameters can be used for efficient transfection [32]: 1) relatively short pulses of 6 × 100 μs [86]; 2) relatively long and low pulses, e.g. 8 × 20 ms [35,80,87-89]; and 3) combination of high-voltage and low-voltage pulses [47,71,90-93]. To use the presented numerical optimization for different pulses, the electroporation thresholds would have to be determined and built into the electroporation models. The presented method of numerical modeling and optimization is generic and can be applied to different electrode configurations (e.g. plate, hexagonal, multi-array), electric pulse parameters (if thresholds *E_rev _*and *E_irr _*are known), other tissues (skin, liver, etc.) or performed for different definition of objective function defined on the basis of specific needs of given electroporation-based clinical applications such as gene therapy, DNA vaccination, electrochemotherapy or ablation by irreversible electroporation.

The presented numerical study of gene electrotransfer is the first study that enables optimization of electric parameters and electrode positions for gene electrotransfer in vivo. Moreover, it takes into account anisotropy of muscle tissue as well as dynamic changes of conductivity during electroporation by using sequential analysis. As such, it presents the first attempt to optimize electric pulse parameters and electrode positioning for gene electrotransfer. Our results can be used as guidelines for researchers and physicians in selecting optimal parameters for in vivo gene electrotransfer and thus indirectly also reduce the number of animals used in clinical studies of gene therapy and DNA vaccination.

## Competing interests

The authors declare that they have no competing interests.

## Authors' contributions

AZ, DM and MP were involved in the design of the study. AZ, SC and MP built the numerical model. AZ performed the parametric study and optimization. AZ and MP performed the data analysis. All authors were involved in the preparing of and have approved the final manuscript.
